# Perioperative redistribution of regional ventilation and pulmonary function: a prospective observational study in two cohorts of patients at risk for postoperative pulmonary complications

**DOI:** 10.1186/s12871-019-0805-8

**Published:** 2019-07-27

**Authors:** Maria Bauer, Anne Opitz, Jörg Filser, Hendrik Jansen, Rainer H. Meffert, Christoph T. Germer, Norbert Roewer, Ralf M. Muellenbach, Markus Kredel

**Affiliations:** 1 Department of Anaesthesia and Critical Care, University Hospital of Würzburg, University of Würzburg, Oberdürrbacher Strasse 6, 97080 Würzburg, Germany; 2 Department of General, Visceral, Transplantation, Vascular and Paediatric Surgery, University Hospital of Würzburg, University of Würzburg, Oberdürrbacher Strasse 6, 97080 Würzburg, Germany; 3 Department of Trauma, Hand, Plastic and Reconstructive Surgery, University Hospital of Würzburg, University of Würzburg, Oberdürrbacher Strasse 6, 97080 Würzburg, Germany

**Keywords:** Electrical impedance tomography, General anaesthesia, Postoperative complications, Pulmonary function tests

## Abstract

**Background:**

Postoperative pulmonary complications (PPCs) increase morbidity and mortality of surgical patients, duration of hospital stay and costs. Postoperative atelectasis of dorsal lung regions as a common PPC has been described before, but its clinical relevance is insufficiently examined. Pulmonary electrical impedance tomography (EIT) enables the bedside visualization of regional ventilation in real-time within a transversal section of the lung. Dorsal atelectasis or effusions might cause a ventral redistribution of ventilation. We hypothesized the existence of ventral redistribution in spontaneously breathing patients during their recovery from abdominal and peripheral surgery and that vital capacity is reduced if regional ventilation shifts to ventral lung regions.

**Methods:**

This prospective observational study included 69 adult patients undergoing elective surgery with an expected intermediate or high risk for PPCs. Patients undergoing abdominal and peripheral surgery were recruited to obtain groups of equal size. Patients received general anesthesia with and without additional regional anesthesia. On the preoperative, the first and the third postoperative day, EIT was performed at rest and during spirometry (forced breathing). The center of ventilation in dorso-ventral direction (COVy) was calculated.

**Results:**

Both groups received intraoperative low tidal volume ventilation. Postoperative ventral redistribution of ventilation (forced breathing COVy; preoperative: 16.5 (16.0–17.3); first day: 17.8 (16.9–18.2), *p* < 0.004; third day: 17.4 (16.2–18.2), *p* = 0.020) and decreased forced vital capacity in percentage of predicted values (FVC%predicted) (median: 93, 58, 64%, respectively) persisted after abdominal surgery. In addition, dorsal to ventral shift was associated with a decrease of the FVC%predicted on the third postoperative day (*r* = − 0.66; *p* < 0.001). A redistribution of pulmonary ventilation was not observed after peripheral surgery. FVC%predicted was only decreased on the first postoperative day (median FVC%predicted on the preoperative, first and third day: 85, 81 and 88%, respectively). In ten patients occurred pulmonary complications after abdominal surgery also in two patients after peripheral surgery.

**Conclusions:**

After abdominal surgery ventral redistribution of ventilation persisted up to the third postoperative day and was associated with decreased vital capacity. The peripheral surgery group showed only minor changes in vital capacity, suggesting a role of the location of surgery for postoperative redistribution of pulmonary ventilation.

**Trial registration:**

This prospective observational single centre study was submitted to registration prior to patient enrollment at ClinicalTrials.gov (NCT02419196, Date of registration: December 1, 2014). Registration was finalized at April 17, 2015.

**Electronic supplementary material:**

The online version of this article (10.1186/s12871-019-0805-8) contains supplementary material, which is available to authorized users.

## Background

Postoperative pulmonary complications (PPCs), defined as atelectasis, pleural effusion, respiratory infection, respiratory failure, pneumothorax, bronchospasm or aspiration pneumonitis, increase morbidity and mortality in surgical patients [1] and lead to longer hospitalization [2] and higher costs [3]. Dorsal atelectasis of about 1–2% of the area of a caudal computed tomography (CT) scan was detected in patients after induction of anesthesia for lower abdominal surgery. These effects persisted during the first postoperative days and were accompanied by reduced forced vital capacity (FVC), forced expiratory volume in one second (FEV1) and impaired arterial oxygenation [4, 5]. Upper abdominal surgery and morbidly obese patients showed even larger areas of atelectasis [4]. However, the actual postoperative redistribution of pulmonary ventilation and its clinical relevance remains unknown. Moreover different groups of patients have not been investigated. To investigate PPCs like atelectasis and pleural effusions the use of CT is limited as it is not available at the bedside and because of the radiation. The restricted time-resolution is another limitation of CT in the examination of dynamic changes during breathing.

Electrical impedance tomography (EIT) is a non-invasive and radiation-free technique that enables the bedside visualization of regional ventilation in real-time within a transversal section of the lungs [6]. It can assess ventilation distribution in critically ill patients during mechanical ventilation and spontaneous breathing [7–9]. EIT was used to optimize the ventilation in animal models of acute respiratory distress syndrome (ARDS) via the titration of positive end-expiratory pressure (PEEP), of morbidly obese patients during laparoscopic surgery and patients with ARDS [10–13]. A ventral redistribution of ventilation was described during mechanical ventilation [14–16] and in the early postoperative period for laparoscopic surgery [15]. The ventral redistribution of ventilation might be a result of dorsal atelectasis or pleural effusions. A recent randomized clinical study used pulmonary EIT to titrate PEEP in order to minimize postoperative atelectasis and overdistension after open and laparoscopic surgery. The usage of EIT resulted in higher PEEP and lower dorsal atelectasis confirmed by CT of the lung after extubation [17]. Studies that examined the change of pulmonary EIT for several days postoperatively in spontaneously breathing patients are lacking. Furthermore, the concomitant use of spirometry would enable the evaluation of lung function together with the effect of deep breathing maneuvers on EIT. Since several operative procedures (e.g. upper abdominal surgery) increase the risk for PPCs [1, 18], an investigation comparing different groups of patients is warranted.

The aim of our study was to examine the perioperative redistribution of regional ventilation and change in FVC in spontaneously breathing patients during their recovery from abdominal and peripheral surgery. We hypothesized a perioperative shift of regional pulmonary ventilation in ventral direction during forced breathing, a decrease of FVC as percentage of predicted values (FVC%predicted) and an association between both.

## Methods

This single centre prospective observational study examined perioperative redistribution of ventilation in abdominal and peripheral surgical patients with expected intermediate to high risk for PPCs. The medical ethics committee of the University Hospital Würzburg, Germany, approved of the study protocol (237/14). All patients provided written informed consent before inclusion.

### Patients

Adult patients (18 years or older) undergoing elective abdominal surgery and peripheral (limb and vessel) surgery were recruited. The patients received general anesthesia with and without additional regional anesthesia such as nerve block or epidural anesthesia. The inclusion of both surgical cohorts allowed us to examine patients with (abdominal) and without (peripheral) direct influence of the site of surgery on postoperative pulmonary function. Exclusion criteria were emergency procedures, re-surgery of hospitalized patients, surgery in local or regional anesthesia alone, expected postoperative ventilation, expected hospital stay less than three days, pregnancy and any contraindication for the application of EIT (e.g. pacemaker). The period of recruitment and follow-up was between January 2015 and February 2016 at the University Hospital of Würzburg. The goal was to recruit patients with expected intermediate (26–44 points) or high risk (> 44 points) for PPCs according to the ARISCAT-Score [1, 18]. Variables of the ARISCAT-score available while screening the patients were: Age, preoperative peripheral oxyhemoglobin saturation (SpO_2_), planned surgical incision and planned duration of surgery. Therefore, we required at least 18 points for the sum of the ARISCAT-variables age (≤50 years: 0, 51–80 years: 3, > 80 years: 16 points), SpO_2_ (≥96%: 0, 91–95%: 8, ≤90%: 24 points), surgical incision (peripheral: 0, upper abdominal: 15, thoracic: 24 points) and planned duration of surgery (< 2 h: 0, 2–3 h: 16, > 3 h: 23 points). After surgery, details about the surgical incision and duration were available to calculate the final ARISCAT-Score together with the remaining variables of the ARISCAT-Score (acute respiratory infection during the previous month, preoperative anemia).

### Study procedures

We performed measurements on the day before surgery, the first and the third postoperative day as follows:

A pulse oximeter applied to a patient’s finger measured SpO_2_ non-invasively before performing spirometry (combined spirometer and pulse oximeter, Spirodoc®, Medical International Research, Roma, Italy).

A silicon belt with 16 equidistant integrated electrodes was applied around the patient’s thorax at the height of the fifth intercostal space in the medioclavicular line. The belt was newly positioned for every measurement at this position. After application of a reference electrode on the abdomen the patient was lying in supine position with a 30 degrees elevated upper body and arms resting besides the upper body. After a stabilization period of two minutes the measurement by EIT (Pulmovista 500**®**, Dräger Medical AG, Lübeck, Germany) was started. First, the tomograph recorded three minutes of spontaneous breathing. Afterwards the pulmonary function was measured by spirometry while EIT recording was continued. The best value of three attempts was used with focus on FVC and FEV1 in percent of predicted values (FVC%predicted and FEV1%predicted) according to Knudson.

Furthermore, we determined the respiratory rate by EIT, pain score by Numeric Rating Scale (0–10) and patient’s dyspnea by questionnaire.

PPCs as defined according to Canet et al. [1] were followed up until the seventh postoperative day or until the patient was discharged from the hospital by examining clinically indicated thoracic x-ray, CT or magnetic resonance tomography and chart review.

The type of anesthesia, airway and intraoperative ventilation were not part of the study protocol, but those data were collected. We calculated the tidal volume in ml per kg predicted body weight. The predicted body weight was calculated according to standard formulas [19].

### Processing of EIT data

PulmoVista 500**®** for EIT recorded changes in thoracic bioimpedance during breathing. Data were analyzed with special software (Dräger EIT Data Analysis Tool 6.1, Dräger Medical AG, Lübeck, Germany). A low pass filter (35 bpm) ensured assessment of ventilation-related impedance changes only. The software used linearized finite elements Newton-Raphson method for transforming EIT data into an image of ellipsoid shape built up by triangular elements. The resulting images were reconstructed into a 32 × 32 pixel matrix and were smoothed by Gaussian function. The EIT image matrix represented the impedance change relative to a baseline. The baseline was set at the expiration of tidal breathing at rest. For evaluation of breathing at rest, the impedance change within the EIT image matrix from baseline to inspiration was averaged over one minute of normal breathing (analysis at rest, minute image). For evaluation of forced breathing, the impedance change within the EIT image matrix from baseline to maximal inspiration before forced expiration during spirometry (analysis at forced breathing, tidal image) was selected. We exported the 32 × 32 matrix of intensity change in % (.ASC File) in Microsoft Excel. This matrix (a_xy_) defined x = 1 (right) to 32 (left) and y = 1 (dorsal) to 32 (ventral). Within this matrix, we calculated a “center of ventilation” (COV) with a sagittal (COVy) and horizontal (COVx) coordinate by using the center of gravity equation according to Luepschen [20]. In similar form, Frerichs [21] initially described this method, which was modified by Radke [14]. COVx: The sum of all entries of the matrix multiplied by their x-coordinate divided by the total sum of the matrix. COVy: The sum of all entries of the matrix multiplied by their y-coordinate divided by the total sum of the matrix:

$$ COVx=\frac{\sum_{x,y=1}^{32}x\times {a}_{x,y}}{\sum_{x,y=1}^{32}{a}_{x,y}}\kern0.96em COVy=\frac{\sum_{x,y=1}^{32}y\times {a}_{x,y}}{\sum_{x,y=1}^{32}{a}_{x,y}} $$

To countenance the summation of EIT images independent of absolute impedance and breathing effort we normalized each matrix of the tidal images to the same global impedance change (arbitrary sum of 32 × 32 intensity changes = 3000%). Afterwards the normalized matrices for each time within a cohort were summated. The summated matrix was also normalized and a colored contour line graph was constructed by graphic software (Origin Pro 9.1 G, OriginLab Corporation, Northhampton, MA, USA). These EIT summation images visualized the change of pulmonary ventilation over a chronological sequence for a whole cohort. To illustrate the distribution of regional ventilation across four ventral to dorsal regions, the relative proportions of total impedance variation in the most dorsal (lines 1–8 of the 32 × 32 matrix), dorsal (lines 9–16), ventral (lines 17–24) and most ventral (lines 25–32) region were calculated.

### Sample size calculation

According to the a priori sample size calculation a change in the quotients of ventilation in ventral to dorsal EIT regions of 0.1 should be detected. This corresponded to a 2.5% sagittal redistribution of ventilation. Our own data showed an estimated standard deviation of the quotients of 0.15. We determined a local α-level of 0.0127 to reach a global α-level of 0.05 according to Sidak-correction for four tests (two comparisons in time, two cohorts). We calculated a sample size of 27 patients in each cohort to show this effect in a two-sided t-test for paired samples with a statistical power of 0.80. We expected a dropout rate of 25% and aimed for up to 36 patients per cohort. The recruitment process ended when *n* = 27 analyzable patients for paired comparison at corresponding time in each cohort were secured.

### Statistical analysis

We defined the primary outcome as change in COVy on the first and third postoperative day compared to the preoperative measurements in the abdominal and peripheral surgery cohort during forced breathing. The secondary outcome was defined as the change in FVC%predicted on the first and third postoperative day compared to preoperative values. We used COVy measured at forced breathing during inspiration for spirometry as primary outcome variable since we tested the association with the results of spirometry.

Several variables did not reach requirements for parametric statistics, therefore we used nonparametric testing and values were shown as median (25th–75th percentile). Comparisons between preoperative and postoperative measurements of COVx and COVy were tested using Wilcoxon signed-rank test and adapted by Sidak adjustment for multiple comparisons (two comparisons in time, two cohorts). Comparisons between forced breathing and breathing at rest were tested with Wilcoxon signed-rank test. Associations between changes of COVy and FVC%predicted, and other variables varying over time was tested using Spearman’s rank correlation. Remaining parameters were tested by Friedman ANOVA for repeated measurements and corrected for multiple testing by Dunn’s Method. Comparisons between both cohorts were tested with Mann-Whitney-U-test and the Fisher exact test. *P*-values < 0.05 in two sided testing were considered statistically significant.

## Results

### Study populations

We included 36 patients undergoing abdominal and 33 patients undergoing peripheral surgery. In the abdominal cohort, two patients underwent re-surgery during the study and were excluded. Four patients refused to participate in postoperative measurements, three of them due to pain or dyspnea, but remained in the study for evaluating PPCs. In the peripheral cohort one patient did not undergo surgery, one received only regional anesthesia and one withdrew consent. Thus, 30 patients of each cohort having at least the preoperative and one postoperative measurement were analyzed regarding EIT and spirometry. Postoperative EIT data were not available due to technical problems or disturbed quality at the first or the third postoperative day. In the abdominal cohort in 4 (first postoperative day) and 3 (third postoperative day) cases and in the peripheral cohort in 1 and 3 cases EIT data were not available. Within the respective cohorts 34 and 30 patients were evaluated for PPCs until discharge or day 7. In the abdominal cohort 10 of 34 patients were discharged between day 3 and 7. In the peripheral cohort 2 of 30 patients were discharged before day 3 and another 15 patients before day 7.

Table 1 depicts patients’ characteristics and details of the surgery and anesthesia. Both groups featured an intermediate risk for PPCs according to the median ARISCAT score. The score was markedly higher in the abdominal cohort, mainly caused by the fact that the patients received upper abdominal surgery in 80%. Furthermore, the duration of surgery and anesthesia was longer compared to the peripheral cohort. On the other hand, patients in the peripheral group were older and had slightly lower preoperative SpO_2_ and hemoglobin concentrations.

**Table 1:** Patients´ characteristics

	Abdominal (*n* = 30)	Peripheral (*n* = 30)	p
Age (years)	65 (56–74)	74 (63–80)	0.034*
Male gender	21 (70%)	20 (67%)	1.000
BMI (kg/m^2^)	27.7 (24.5–32.0)	26 (24.5–28.7)	0.156
ASA physical status	1: 0 (0%)	1: 0 (0%)	0.089
	2: 17 (57%)	2: 10 (33%)	
	3: 12 (40%)	3: 19 (63%)	
	4: 1 (3%)	4: 1 (3%)	
Respiratory infection last month	7 (23%)	8 (27%)	1.000
Preoperative stay (days)	1 (1–1)	1 (1–2)	0.009*
Preoperative SpO_2_ (%)	97 (96–98)	96 (95–97)	0.022*
Preoperative Hb (g/dl)	13.3 (12.3–14.3)	12.7 (11.0–13.3)	0.021*
Anesthesia	general: 7 (23%)	general: 28 (93%)	
	gen. + epidural: 23 (77%)	gen. + nerve block: 2 (7%)	
Duration of surgery (minutes)	258 (171–343)	163 (120–208)	< 0.001*
Duration of anesthesia (minutes)	333 (236–394)	220 (170–250)	< 0.001*
Surgical incision	lower abdomen: 6 (20%)	peripheral: 30 (100%)	< 0.001*
	upper abdomen: 24 (80%)		
ARISCAT score	41 (34–43)	30 (19–38)	0.007*

*ASA* American Society of Anesthesiologists, *BMI* Body mass index, *SpO*_*2*_ oxyhemoglobin saturation by pulse oximetry breathing room air in supine position, Hb = hemoglobin concentration, ARISCAT = risk score for postoperative pulmonary complications [1, 18]. Data are median (25th–75th percentile), *p < 0.05 (Mann-Whitney-U-test and Fisher exact test)

Types of surgery in the abdominal cohort were interventions on the liver (*n* = 13; 38%), rectum (*n* = 6; 18%), sigmoid colon (*n* = 2; 6%), colon (*n* = 5; 15%), pancreas (*n* = 2; 6%), spleen (*n* = 1; 3%), gastric surgery (*n* = 2; 6%), cholecystectomy (*n* = 2; 6%), and explorative laparotomy (*n* = 1; 3%). In the peripheral surgery cohort 15 patients underwent limb surgery and 15 vessel surgery.

Patients of both cohorts received low tidal volume ventilation. Essential ventilation parameters as tidal volume, maximal PEEP and inspiratory pressure were comparable between both groups (Additional file 1: Table S1).

### Postoperative findings in the abdominal surgery cohort

**Fig. 1:**
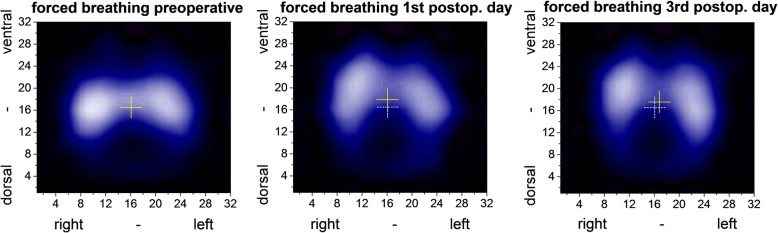
Electric impedance tomography summation images during forced breathing in the abdominal surgery cohort over time. Summation images of normalized tidal images in 30 (preoperative), 26 patients (first postoperative day) and 27 patients (third postoperative day) are shown. The images depict changes from normal expiration to maximal inspiration. The center of the cross represents the center of ventilation (COV). Postoperatively the full cross represents the actual COV and the dashed cross the preoperative COV

**Fig. 2:**
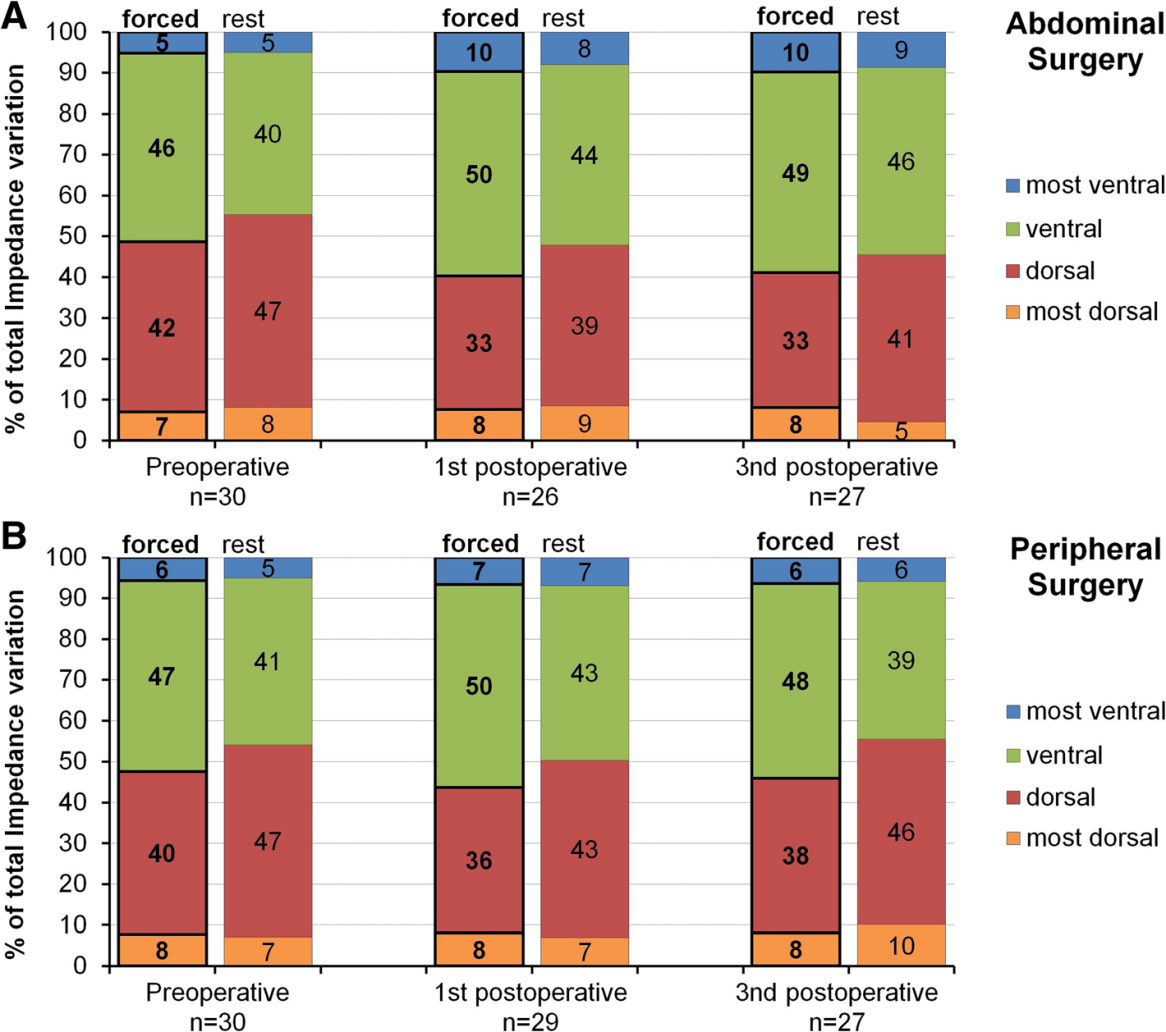
Distribution of regional ventilation during forced breathing and breathing at rest within four dorsal to ventral regions of equal size. **a** Abdominal surgical cohort. **b** Peripheral surgical cohort. Percentages of total impedance variation in electric impedance tomography represent the regional ventilation in the most dorsal (lines 1–8 of the 32 × 32 matrix), dorsal (lines 9–16), ventral (lines 17–24) and most ventral (lines 25–32) regions preoperatively, as well as during the first postoperative and third postoperative day

The COV describes the distribution of ventilation by a horizontal (COVx) and a sagittal (COVy) coordinate as illustrated by Fig. 1. COVy underwent a perioperative shift from dorsal to ventral during forced breathing (first postoperative day: *p* < 0.004; third postoperative day: *p* = 0.020) (Table 2, Fig. 1). Concomitantly, pulmonary ventilation shifted to the left side (COVx) on the third postoperative day. Figure 2a shows the distribution of ventilation in four dorsal to ventral regions in the abdominal surgery cohort. The percentage of ventilation in the ventral and most ventral regions increased postoperatively while the dorsal regions received less ventilation.

**Table 2:** Ventilation distribution and respiratory parameters of patients undergoing abdominal surgery

	Preoperative (*n* = 30)	1st postop. Day (*n* = 29)	3rd postop. Day (*n* = 29)
COVy			
forced	16.5 (16.0–17.3)	17.8 (16.9–18.2)#	17.4 (16.2–18.2)#
at rest	16.3 (14.5–17.2)$	17.1 (15.5–17.9)$	17.3 (15.8–18.2)
COVx			
forced	16.1 (15.9–16.4)	16.1 (15.8–16.5)	16.8 (16.1–17.4)#
at rest	15.7 (15–16.3)$	15.7 (14.8–16.4)$	16.4 (15.9–17.3)#
FVC%predicted	93 (80.3–106)	58 (49.5–64.5)*	64 (45.5–77.3)*
FEV1%predicted	91 (79–98)	53 (45–65)*	61 (46–73)*
Tiffeneau index	0.74 (0.71–0.78)	0.78 (0.73–0.81)*	0.77 (0.75–0.79)*
SpO_2_%	97 (96–98)	93 (90–95)*	93 (91–96)*
Respiratory rate /min	14 (12–16)	18 (14–19)	16 (13–18)
Pain NRS			
forced	0 (0–0)	3 (1–5)*	1 (0–2)*
at rest	0 (0–0)	1 (0–3)*	0 (0–2)*
Epidural analgesia		23 (79%)	21 (72%)
Respiration			
room air	30 (100%)	15 (52%)	27 (90%)
supplemental oxygen	0 (0%)	14 (48%)	2 (7%)
*mechanical ventilation*	*0*	*1*	*0*
Dyspnea			
no	28 (93%)	26 (90%)	24 (83%)
slight	2 (7%)	2 (7%)	4 (14%)
moderate	0 (0%)	1 (3%)	0 (0%)
severe	0 (0%)	0 (0%)	1 (3%)

FVC%predicted = % of predicted forced vital capacity, FEV1%predicted = % of predicted forced expiratory volume in one second, COVx = Center of Ventilation horizontal axis, COVy = Center of Ventilation vertical axis, COV values are coordinates of a 32 × 32 matrix, SpO_2_ = oxyhemoglobin saturation by pulse oximetry breathing room air in supine position, NRS = numeric rating scale (0–10). Data are median (25th–75th percentile). *p < 0.05 versus preoperative (Friedmann ANOVA, Dunns post hoc test). #*p* < 0.05 versus preoperative (Wilcoxon signed rank test, Sidak correction). $p < 0.05 versus forced (Wilcoxon signed rank test)

**Fig. 3:**
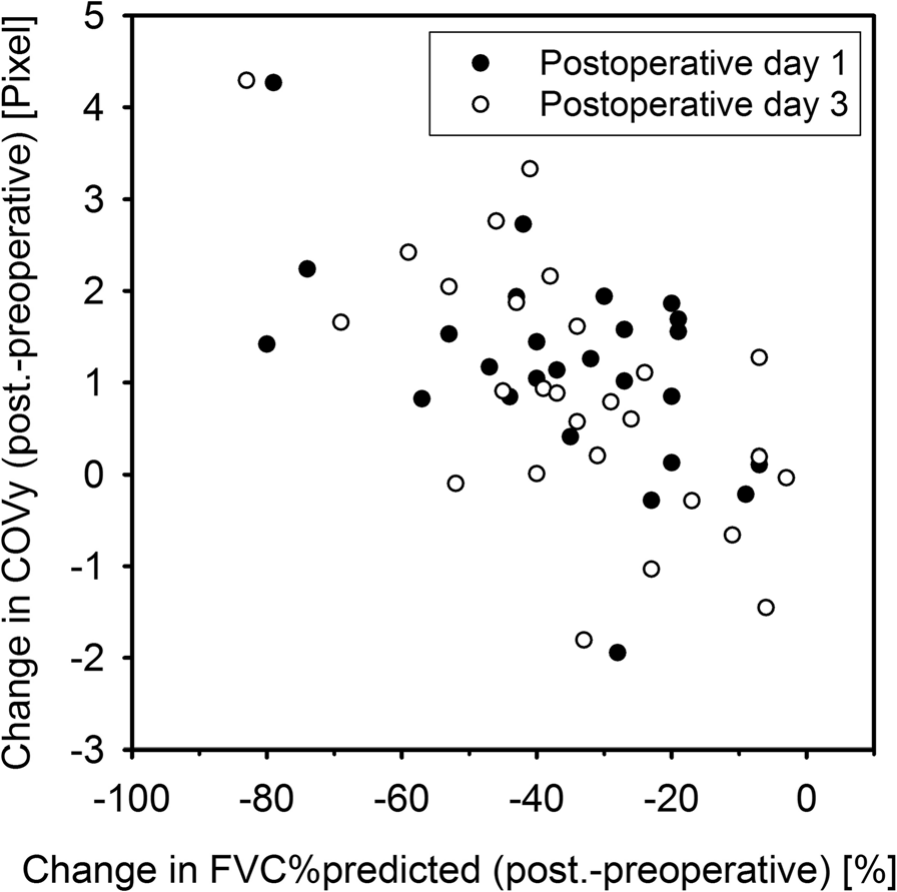
Association between perioperative changes in COVy and FVC%predicted during forced breathing. A scatter plot for the changes in COVy and FVC%predicted on the first (full circles) and on the third postoperative day (empty circles) is shown. Changes from preoperative to postoperative measurements were used for both variables

FVC%predicted was decreased postoperatively (Table 2). There was a relevant within-patient correlation for changes in FVC%predicted (postoperative-preoperative values) between the first and third postoperative day (*r* = 0.732; *p* < 0.001). Hence, correlation tests with changes in FVC%predicted were separately performed for both postoperative days. Perioperative changes in COVy were negatively associated with changes in FVC%predicted (Fig. 3) while forced breathing (*r* = − 0.66; *p* < 0.001) on the third postoperative day. Statistical significance was not reached on the first postoperative day (*r* = − 0.37, *p* = 0.064).

SpO_2_ at room air decreased postoperatively. Perioperative respiratory rate remained stable. Postoperative pain levels increased (Table 2). The changes of COVy were weakly associated with postoperative pain levels at forced breathing (*r* = 0.291, *p* = 0.034). There was no association between changes of COVy and SpO_2_ at forced breathing (*r* = − 0.175, *p* = 0.208) and breathing at rest (*r* = − 0.253, *p* = 0.068). PPCs occurred in 10 out of 34 patients as pleural effusion (*n* = 8), respiratory failure (*n* = 5) and atelectasis (*n* = 2).

### Postoperative findings in the peripheral surgery cohort

Neither forced breathing nor breathing at rest led to a statistically significant perioperative redistribution of pulmonary ventilation as measured by COVy (Table 3). The distribution of ventilation between four dorsal to ventral lung regions remained similar throughout the perioperative course (Fig. 2b).

**Table 3:** Ventilation distribution and respiratory parameters of patients undergoing peripheral surgery

	Preoperative (*n* = 30)	1st postop. Day (*n* = 30)	3rd postop. Day (*n* = 28)
COVy			
forced	16.6 (15.5–17.1)	16.7 (16.3–17.4)	16.6 (15.8–17.2)
at rest	16.1 (14.9–17.1)$	16.6 (15.8–17.3)	15.5 (14.7–17)$
COVx			
forced	16.2 (15.8–16.6)	16.2 (15.8–16.7)	16.2 (15.8–16.7)
at rest	15.7 (15–16.2)$	15.7 (15.1–16)$	15.7 (15–16.4)$
FVC%predicted	88 (64–98)	82 (65–95)*	94 (74–99)
FEV1%predicted	85 (54–98)	81 (63–94)*	88 (64–105)
Tiffeneau index	0.75 (0.64–0.81)	0.79 (0.69–0.81)	0.77 (0.73–0.81)
SpO_2_%	96 (94–97)	95 (93–96)*	96 (95–97)
Respiratory rate /min	14 (12–16)	17 (14–18)*	16 (12–19)
Pain NRS			
forced	0 (0–0)	0 (0–2)	0 (0–0.5)
at rest	0 (0–3)	2 (1–4)*	1 (0–2)
Continuous nerve block		0 (0%)	0 (0%)
Respiration			
room air	29 (97%)	25 (83%)	25 (89%)
supplemental oxygen	1 (3%)	5 (17%)	3 (11%)
*mechanical ventilation*	*0*	*0*	*0*
Dyspnea			
no	26 (87%)	29 (97%)	26 (93%)
slight	3 (10%)	1 (3%)	2 (7%)
moderate	1 (3%)	0 (0%)	0 (0%)
severe	0 (0%)	0 (0%)	0 (0%)

FVC%predicted = % of predicted forced vital capacity, FEV1%predicted = % of predicted forced expiratory volume in one second, COVx = Center of Ventilation horizontal axis, COVy = Center of Ventilation vertical axis, COV values are coordinates of a 32 × 32 matrix, SpO_2_ = oxyhemoglobin saturation by pulse oximetry breathing room air in supine position, NRS = numeric rating scale (0–10). Data are median (25th–75th percentile). *p < 0.05 versus preoperative (Friedmann ANOVA, Dunns post hoc test). $*p* < 0.05 versus forced (Mann-Whitney-Rank Sum Test)

Median FVC%predicted was decreased on the first postoperative day by about 6% (Table 3). Changes in COVy were neither associated with changes in FVC%predicted while forced breathing (*r* = − 0.081; *p* = 0.55) nor during breathing at rest (*r* = − 0.204; *p* = 0.13).

Only on the first postoperative day SpO_2_ was slightly decreased, respiratory rate and pain score at rest were increased (Table 3). PPCs occurred in 2 out of 30 patients (respiratory failure).

### Difference in regional ventilation during forced breathing and breathing at rest

During forced breathing COV redistributed in ventral direction (COVy) and on the left side (COVx) compared to breathing at rest in the abdominal (Table 2) and in the peripheral surgery group (Table 3) at several time points. Figure 2a and b illustrate that ventral regions received more ventilation during forced breathing compared to breathing at rest, while the dorsal regions received less.

## Discussion

Our study is the first that investigated two representative cohorts of spontaneously breathing patients with EIT and spirometry up to three days postoperatively. We showed a postoperative ventral redistribution of ventilation according to EIT and decreased FVC%predicted in patients after abdominal surgery. These findings persisted up to the third postoperative day. The dorsal to ventral shift during forced breathing was associated with a decrease in FVC%predicted, suggesting that impaired aeration of the dorsal parts of the lung had a negative impact on pulmonary capacity. Patients after peripheral surgery did not show a redistribution of pulmonary ventilation. In this cohort FVC%predicted was decreased only on the first postoperative day. Presumably, the site of surgical incision was more important for changes in ventilation than the influence of anesthesia in patients with elevated risk for postoperative pulmonary complications.

Impaired aeration of the dorsal lung in the abdominal group might be a result of postoperative dorsal atelectasis and pleural effusions. However, no planned serial radiographic examinations were performed. Postoperative atelectasis of dependent lung regions persisting for days was found by CT studies [4, 5] and the extent of atelectasis was negatively correlated with forced vital capacity [5]. However, low extent of atelectasis depicted in CT scans did not explain the marked postoperative impairment in forced vital capacity [5, 22]. In contrast to CT imaging EIT visualizes pulmonary ventilation. Several studies using EIT revealed a ventral redistribution of pulmonary ventilation after starting mechanical ventilation [14, 16, 21, 23]. Schaefer [23] and Radke [14] with colleagues showed that redistribution immediately reversed after postoperative weaning from mechanical ventilation. Both laparoscopic and open cholecystectomy decreased postoperative pulmonary function measured by spirometry whereas laparoscopic technique showed less impairment [24]. Treschan et al. showed impaired pulmonary function after both high and low tidal volume ventilation up to the fifth day after upper abdominal surgery [25]. Examinations by EIT that followed up on pulmonary ventilation and simultaneously tested pulmonary function via spirometry several days postoperatively in patients with elevated risk for PPCs were lacking. Our study was able to show ongoing pulmonary dysfunction in the abdominal cohort at least until the third postoperative day. The sample size was sufficient according to a priori power calculation.

Impaired pulmonary function in terms of decreased FVC%predicted on the first postoperative day after peripheral surgery was probably a consequence of general anesthesia. An influence of the surgical incision site can be excluded. The rapid recovery of pulmonary function and the non-redistribution of pulmonary ventilation facilitate this hypothesis. Postoperative pain levels increased after peripheral surgery only at rest but not during forced breathing.

Both cohorts were not comparable in their characteristics. A comparison of outcome variables between both cohorts was not planned. The observed results after abdominal surgery were possibly caused by patients´ characteristics. This is reflected by the different values of the ARISCAT score. We cannot exclude that patients undergoing peripheral surgery featuring a higher ARISCAT score would show the same postoperative changes in spirometry and EIT. In order to allow a meaningful group comparison a matched pairs analysis between the cohorts is necessary. However, such an analysis of our data would result in a lower number of cases so that sufficient statistical power would not be reached.

Surgical incision and duration of surgery mainly accounted for the differences between both cohorts regarding ARISCAT score and the risk for PPCs. Other differences, such as age, SpO_2_ and hemoglobin concentration were less marked and had a lower influence on the risk for PPCs. If we excluded the influence of surgical incision site from the ARISCAT score, the score would be 26 (24–28) in the abdominal group, which is comparable to the peripheral group. From this perspective, both groups featured a similar risk for PPCs except the site of surgical incision. Therefore, the ventral redistribution of ventilation and reduced FVC%predicted might be a result of the localization of the surgical incision. Postoperative diaphragmatic dysfunction after upper abdominal surgery leads to a reduced ventilation and expansion of the basal parts of the lungs [26]. This could explain the findings, since 80% of the patients in the abdominal group received upper abdominal surgery. Therefore, ventral redistribution of ventilation and decreased FVC%predicted are explainable through reduced recruitment of the dorsal lung after abdominal surgery. As described, patients in the peripheral surgery group were markedly older. According to a recent study of oxygenation impairment during anesthesia, poorly ventilated but perfused lung regions increasing with age mainly contributed to the impaired arterial oxygenation in the elderly patients. In contrast, pulmonary shunt fraction in nonventilated lung regions correlated with atelectasis shown by chest CT and was highest in middle-aged patients. [27]. Hence, a higher susceptibility to atelectasis in the younger patients of the abdominal group of the present study might have also contributed to the ventral redistribution of ventilation.

By positioning the EIT belt at the height of the fifth intercostal space in the medioclavicular line dorso-basal parts of the lung are represented in the EIT images. Furthermore, previous studies showed that this belt position is appropriate for examining both mechanically ventilated and spontaneously breathing patients [28, 29]. Higher pain levels during forced breathing in patients that underwent abdominal surgery could explain postoperative shallow breathing leading to preferred ventilation of the ventral lung. Nevertheless, most patients undergoing abdominal surgery received an epidural catheter, most likely preventing a significant influence of pain. However, during forced breathing ventilation was relocated from dorsal to ventral regions at several time points in both groups of our study. That finding indicates that deeper inspiration recruited more ventral regions of the lung. Thus, more shallow breathing would not explain postoperative relocation to ventral and most ventral lung regions in the abdominal group. Respiratory rate was not markedly elevated in the abdominal group, also arguing against an effect caused by shallow breathing. Dorso-basal effusions and atelectasis in the abdominal cohort would also explain lower postoperative SpO_2_ that contributed to the higher count of PPCs in this group.

EIT is a suitable technique for investigating postoperative redistribution of ventilation. Through its non-invasive and radiation-free characteristics combined with a good reproducibility of repeated EIT measurements [30], it is a good method for close pulmonary monitoring. The exact positioning of the EIT belt according to anatomical landmarks as done in the present study is necessary to ensure a good reproducibility of EIT images.

The present study is limited by the fact that EIT was only applied in one plane of the chest. Measurements at additional planes were not made to limit the burden for the patients after major surgery. In addition, there were only two postoperative measurements and the changes of COVy and FVC%predicted found in the abdominal surgery group were ongoing at the third postoperative day. Hence, the behavior of both outcome variables and their relation during full recovery remains unclear. A further limitation is that the study solely relies on EIT as imaging method. Serial CT scans during this study would provide additional information about the amount of atelectasis and effusion in the dorsal lung as CT scans are very sensitive for detecting atelectasis [5]. However, imaging the distribution of lung ventilation by EIT and CT does not correlate well, since both methods depict different features of the lung. EIT shows the actual air flow within the lung, not lung morphology [31]. Therefore, our work adds new information to this field in addition to perioperative studies using CT.

## Conclusions

After abdominal surgery with intraoperative low tidal volume ventilation a ventral redistribution of ventilation during forced breathing and a decreased forced vital capacity persisted until the third postoperative day. Postoperative ventral redistribution of ventilation was associated with a decrease in forced vital capacity. After peripheral surgery, those changes were not evident, despite the risk for postoperative pulmonary complications without consideration of the surgical incision site being comparable to the abdominal group. Besides other characteristics of the patients, the site of surgery might have promoted the pulmonary impairment in patients after abdominal surgery.

## Additional file

Additional file 1: **Table S1.** Ventilation Parameters. Compares the settings of the ventilator throughout anesthesia in both groups. (DOCX 19 kb)

## Data Availability

The datasets used and/or analyzed during the current study are available from the corresponding author on reasonable request.

## Abbreviations

ARDS: Acute respiratory distress syndrome
COVx: Center of ventilation, horizontal coordinate
COVy: Center of ventilation, sagittal coordinate
EIT: Electrical impedance tomography
FEV1: Forced expiratory volume in one second
FVC%predicted: Forced vital capacity in percentage of predicted values
PEEP: Positive end-expiratory pressure
PPC: Postoperative pulmonary complication
SpO_2_: Peripheral oxyhemoglobin saturation
