# Supplementary material for: Perioperative redistribution of regional ventilation and pulmonary function: a prospective observational study in two cohorts of patients at risk for postoperative pulmonary complications
Source: BMC Anesthesiol. 2019 Jul 27;19:132. doi: 10.1186/s12871-019-0805-8 (PMC6661098; doi:10.1186/s12871-019-0805-8)
Supplement: Supplementary file 1 — Table S1. Ventilation Parameters. Compares the settings of the ventilator throughout anesthesia in both groups. (DOCX 19 kb) [file 12871_2019_805_MOESM1_ESM.docx]

**Additional file 1: Table S1: Ventilation parameters**

|  |  | Abdominal (n=30) | Peripheral (n=30) | P |
| --- | --- | --- | --- | --- |
| PEEP (cmH_2_O) | min | 4 (4 - 5) | 5 (4 - 5) | 0.024* |
|  | max | 5 (5 – 8) | 5 (5 - 7) | 0.528 |
| Pinsp (cmH_2_O) | min | 12 (11 - 14) | 13 (11 - 15) | 0.396 |
|  | max | 18 (15 - 21) | 16 (14 - 19) | 0.155 |
| TV (ml/kg) | min | 6.3 (5.9 - 6.9) | 6.4 (5.9 - 7.0) | 0.762 |
|  | max | 7.6 (7.0 - 8.5) | 7.4 (6.9 - 8.3) | 0.608 |
| ExpCO_2_ (mmHg) | min | 33 (31 - 34) | 33 (32 - 34) | 0.266 |
|  | max | 37 (36 - 39) | 36 (36 - 37) | 0.468 |
| FiO_2_ | min | 0.33 (0.30 - 0.37) | 0.39 (0.35 - 0.41) | 0.015* |

PEEP=positive end-expiratory pressure, Pinsp=inspiratory pressure, TV=tidal volume, ExpCO_2_=end-expiratory CO_2_, FiO_2_= fraction of inspired oxygen. min=minimal and max=maximal values during anesthesia are shown as median (25^th^-75^th^ percentile). *p<0.05 (Mann-Whitney-U-test).
